# Th1/17-Biased Inflammatory Environment Associated with COPD Alters the Response of Airway Epithelial Cells to Viral and Bacterial Stimuli

**DOI:** 10.1155/2019/7281462

**Published:** 2019-08-25

**Authors:** Yifan Chen, Rakesh K. Kumar, Paul S. Thomas, Cristan Herbert

**Affiliations:** ^1^Mechanisms of Disease and Translational Research, School of Medical Sciences, UNSW Sydney, Sydney 2052, Australia; ^2^Prince of Wales Clinical School, Faculty of Medicine, UNSW Sydney, Sydney 2052, Australia; ^3^Department of Respiratory Medicine, Prince of Wales Hospital, Sydney 2031, Australia

## Abstract

Chronic obstructive pulmonary disease (COPD) is characterized by airway inflammation associated with a Th1/17-biased cytokine environment. Acute exacerbations of COPD (AECOPD) are most often triggered by respiratory infections, which elicit an exaggerated inflammatory response in these patients, via poorly defined mechanisms. We investigated the responses of airway epithelial cells (AECs) to infective stimuli in COPD and the effects of the Th1/17-biased environment on these responses. Cytokine expression was assessed following exposure to virus-like stimuli (poly I:C or imiquimod) or bacterial LPS. The effects of pretreatment with Th1/17 cytokines were evaluated in both primary AECs and the Calu-3 AEC cell line. We found that poly I:C induced increased expression of the proinflammatory cytokines IL1*β*, IL6, CXCL8, and TNF and IFN-*β*1 in AECs from both control subjects and COPD patients. Expression of IL1*β* in response to all 3 stimuli was significantly enhanced in COPD AECs. Primary AECs pretreated with Th1/17 cytokines exhibited enhanced expression of mRNA for proinflammatory cytokines in response to poly I:C. Similarly, Calu-3 cells responded to virus-like/bacterial stimuli with increased expression of proinflammatory cytokines, and a Th1/17 environment significantly enhanced their expression. Furthermore, increased expression of pattern recognition receptors for viruses (TLR3, TLR7, IFIH1, and DDX58) was induced by Th1/17 cytokines, in both primary AECs and Calu-3 cells. These findings suggest that the Th1/17-biased environment associated with COPD may enhance the proinflammatory cytokine response of AECs to viral and bacterial infections and that increased signaling via upregulated receptors may contribute to exaggerated inflammation in virus-induced AECOPD.

## 1. Introduction

Chronic obstructive pulmonary disease (COPD) is a common, usually progressive inflammatory disease of the airways, which is characterized by persistent airflow obstruction. Risk factors for COPD include a combination of genetic predisposition and long-term exposure to various noxious particles or gases, most importantly cigarette smoke. Worldwide, COPD affects over 250 million people [1] and is expected to be the 3^rd^ leading cause of death by 2020 [2]. Patients with COPD may experience acute exacerbations of their disease (AECOPD) characterized by exaggerated inflammation and worsening of symptoms. Respiratory infections with viruses and bacteria are the most common triggers for AECOPD, accounting for approximately 75% of exacerbations [3]. In these patients, the inflammatory response to respiratory infections is significantly greater than in those without COPD [4]. However, the mechanisms underlying this exaggerated inflammatory response in AECOPD are still poorly understood.

Airway epithelial cells (AECs) play a key role in the pathogenesis of COPD. These cells are the first sites of contact for inhaled environmental stimuli such as cigarette smoke, and they can also contribute to host defense to those irritants [5]. In addition, AECs are the site of infection and replication for many respiratory pathogens. Infections are likely to amplify the inflammatory responses in COPD and consequently worsen the disease, especially via the release of cytokines by AECs. This AEC-derived cytokine response could be altered by the local inflammatory environment in COPD, which is characterized by increased numbers of T helper type 1 and type 17 (Th1 and Th17) cells [6, 7]. Infiltration of Th1/17 cells leads to the accumulation of Th1/17-derived cytokines in the airways, predominantly IFN-*γ* and IL17A. However, the effects of this Th1/17-biased inflammatory environment associated with COPD on the behavior of AECs remain poorly defined.

The local cytokine environment has been shown to modulate inflammatory responses in allergic asthma, another chronic disease of the airways, which is associated with Th2-driven allergic airway inflammation [8]. In the Th2 cytokine environment, characterized by increased levels of IL4 and IL-13, we have previously shown that there is significant enhancement of the airway epithelial response to a viral stimulus, with increased expression of both proinflammatory cytokines (e.g., CXCL8 and IL6) and antiviral response genes (including IFN-*β*1 and IFN-*λ*2/3) [9]. Thus, understanding of the effect of the Th1/17-biased cytokine environment associated with COPD on the response of AEC to infections is of considerable interest, as it may help to explain the exaggerated inflammatory response in virus/bacteria-induced AECOPD.

In this study, we examined whether a Th1/17-biased inflammatory environment altered the *in vitro* response of AEC to viral and bacterial components, to which the cells would be exposed during infective exacerbations of COPD. We first compared cytokine production by AECs from patients with cigarette smoke-induced COPD and healthy controls in response to bacterial and viral stimuli. Then, we established an *in vitro* culture system with the cell line Calu-3, to simulate the Th1/17-biased milieu of COPD, and assessed the appropriateness of this model based on the observations from the primary AECs. Using this model, we investigated how the Th1/17-biased cytokine environment associated with COPD alters the phenotype of AECs and regulates airway epithelial responses to bacterial and viral infections, both in terms of cytokine production and the expression of pattern recognition receptors.

## 2. Material and Methods

### 2.1. Culture of Primary AECs and Calu-3 Cells

Primary human bronchial epithelial cells (HBECs) (passage 1) were purchased from Lonza (Lonza Australia, Melbourne, Australia). These cells were isolated from the epithelial lining of airways of 7 healthy nonsmoking controls (normal human bronchial airway epithelial cells; NAECs) and from 7 deceased patients with cigarette smoke-induced COPD (COPD human bronchial airway epithelial cells; CAECs) whose lung tissues were donated to Lonza.

Primary AECs were cultured in T75 flasks (Corning, NY, USA) in serum-free bronchial epithelial cell growth medium (BEGM, Lonza), comprised of bronchial epithelial cell basal medium (BEBM, Lonza) and a bullet kit (Lonza Australia) containing bovine pituitary extract, insulin, hydrocortisone, retinoic acid, transferrin, gentamicin sulfate/amphotericin, epinephrine, and recombinant human epidermal growth factor (rhEGF). Cells were grown with 5% CO_2_ at 37°C.

In other experiments, the well-differentiated and characterized human airway epithelial cell line, Calu-3 (HTB-55, American Type Culture Collection, Manassas, VA, USA), was used. Cells were cultured in T75 flasks in minimum essential medium (Gibco, Carlsbad, CA, USA) containing 10% heat-inactivated fetal bovine serum (Sigma-Aldrich), 1% L-glutamine, 1% sodium pyruvate, and 1% penicillin/streptomycin at 37°C in an atmosphere of 5% CO_2_.

### 2.2. Pretreatment and Stimulation of Primary AECs and Calu-3 Cells

Primary AECs (passage 2 or passage 3) were seeded in 6-well plates at a density of 2 × 10^5^ cells/well in 2 ml BEGM. To assess responses to virus-like stimuli, AECs were stimulated with 1 *μ*g/ml of a synthetic double-stranded RNA (dsRNA) poly I:C (InvivoGen, San Diego, CA) or with 7 *μ*g/ml imiquimod (InvivoGen), a toll-like receptor (TLR) 7 agonist in basal media (i.e., without other supplements or antibiotics). To assess responses to a bacterial antigen, cells were stimulated with 1.5 *μ*g/ml lipopolysaccharide (LPS) from *Escherichia coli* serotype O111:B4 (Sigma-Aldrich, St. Louis, MO, USA) in basal media. After 4 hours, cells were harvested by lysis with TRI Reagent (Sigma-Aldrich) for isolation of total RNA. At 24 hours, culture supernatants were collected and stored at −20°C for protein assays.

In order to model the Th1/17 cytokine environment of COPD, primary human airway epithelial cells (passage 4 or 5) were grown in the presence or absence of IFN-*γ* and IL17A (PeproTech, Rehovot, Israel). Cells were seeded in 6-well plates in BEGM either with or without human IFN-*γ* and IL17A (both at 6 ng/ml) for 48 hours, of which the last 24 hours was in basal media with 1% complete growth media. Cells were then stimulated with either 1 *μ*g/ml poly I:C, 7 *μ*g/ml imiquimod, or 1.5 *μ*g/ml LPS in basal media for 4 hours.

To assess the effects of Th1/17 cytokine pretreatment on the responses of AEC to virus/bacteria-like stimuli, Calu-3 cells were seeded in 6-well plates at a density of 2 × 10^5^ cells/well in 2 ml of media in the presence or absence of 2 ng/ml of human IFN-*γ* and IL17A for 48 hours, of which the last 24 hours was in medium containing 0.1% FBS. Cells were then stimulated with either poly I:C (10 *μ*g/ml), imiquimod (10 *μ*g/ml), or LPS (500 ng/ml) for 4 h in serum-free media. Cells cultured in medium alone were used as negative controls. Culture supernatants were collected and stored at -20°C while cells were lysed in TRI Reagent for isolation of RNA. Five independent experiments were performed.

The concentrations of all cytokines and stimuli used in this study were determined based on data from previous titration experiments (Supplementary Material file; [Supplementary-material supplementary-material-1]). At the selected concentrations, AECs demonstrated moderate baseline responses to cytokine pretreatments. The duration of pretreatment and stimulation was selected based on the protocols established in our previous study [9].

### 2.3. RNA Isolation and Reverse Transcription

Cells were harvested and lysed in 1 ml TRI Reagent. Total RNA was isolated according to the manufacturer's recommendations and dissolved in RNase-free water (Qiagen, VIC, Australia). The purity and concentration of isolated RNA was assessed using a Nano-drop spectrophotometer (Thermo Scientific, NSW, Australia). RNA samples with a purity ratio (A260/A280) of 1.8~2 were considered as acceptable. Samples were stored at -20°C (short term) or -80°C (longer term) prior to reverse transcription.

RNA was treated with DNase (Invitrogen, NSW, Australia) to remove genomic DNA from the samples prior to reverse transcription. DNase-treated RNA (1 *μ*g total RNA) was then reverse-transcribed using the Transcriptor First Strand cDNA Synthesis Kit (Roche, NSW, Australia) according to the manufacturer's instructions. The resultant cDNA samples were diluted in distilled water and stored at -20°C until use.

### 2.4. Expression of mRNA

Quantitative real-time PCR was used to assess the expression of proinflammatory cytokine genes, including *IL1β*, *IL6*, *CXCL8*, and *TNF*, *IFNB1*, and the pattern recognition receptors *TLR3*, *TLR4*, and *TLR7* (toll-like receptors for viruses and bacteria) as well as *DDX58* and *IFIH1* (RIG-I-like receptors (RLRs) for viruses). Amplified products were detected using SYBR green (BioLine, Taunton, MA, USA). Primers were designed in-house (Table 1). All primer pairs have passed the BLAST specificity screen (specifically bind to corresponding target gene). Reactions were performed using a LightCycler 480 (Roche Diagnostics, Indianapolis, IN, USA), with gene expression normalized to the housekeeping gene hypoxanthine-guanine phosphoribosyl-transferase (HPRT). All samples were assessed in triplicate.

Additional details of the methods for RNA isolation, cDNA synthesis, and real-time PCR are presented in the Supplementary Material file; [Supplementary-material supplementary-material-1].

### 2.5. Protein Immunoassays

The concentrations of IL1*β*, IL6, CXCL8, and TNF in the culture supernatants were determined using enzyme-linked immunoassays (R&D Systems, Minneapolis, USA) according to the manufacturer's instructions. Each sample was assessed in duplicate. The effective assay ranges of ELISA kits are listed in Table 2.

### 2.6. Statistical Analysis

Data are presented as arithmetic means ± SEM. For evaluation of responses to the virus-like/bacterial stimuli, which involved multiple comparisons between 3 treatment groups and a control group, one-way or repeated measures two-way ANOVA was used as appropriate, followed by a Holm-Sidak posttest, with logarithmic transformation of mRNA expression data. *t-*tests were used where comparisons only involved two groups. Specifically, for comparisons between NAEC and CAECs, unpaired *t-*tests were performed, with logarithmic transformation of mRNA expression data. For comparisons between cells cultured in the absence or presence of Th1/17 cytokines, ratio paired *t*-tests or unpaired *t*-tests were used, as appropriate. For clarity, all mRNA data were analyzed using expression value relative to the housekeeping gene HPRT. Data in some graphs are presented as mean fold change in expression relative to the corresponding unstimulated control. The software package GraphPad Prism 7.03 (GraphPad Software, San Diego, CA, USA) was employed for data analysis and preparation of graphs. A *p* value of <0.05 was considered statistically significant.

## 3. Results

### 3.1. Patient Characteristics

Primary epithelial cells were obtained from 7 healthy subjects (NAEC) and 7 patients with COPD (CAEC) (Table 3). Both the control and patient groups comprised 4 females and 3 males, with mean ages of 55.4 ± 4.1 and 58.7 ± 5.5 years, respectively (*p* = 0.43). All subjects in the control group were nonsmokers while all patients with COPD had a long-term smoking history (31 ± 10.3 years).

### 3.2. Epithelial Responses to Virus-Like/Bacterial Stimuli in COPD

AECs derived from patients with COPD and healthy subjects were stimulated with poly I:C (ligand for TLR3), imiquimod (ligand for TLR7), or LPS (bacterial endotoxin, ligand for TLR4). To characterize the responses to these stimuli, the expression of proinflammatory cytokines/chemokines and interferon-*β*1 by the cells was assessed using qRT-PCR. Production of proinflammatory cytokines/chemokines was confirmed by ELISA.

**Figure 1:**
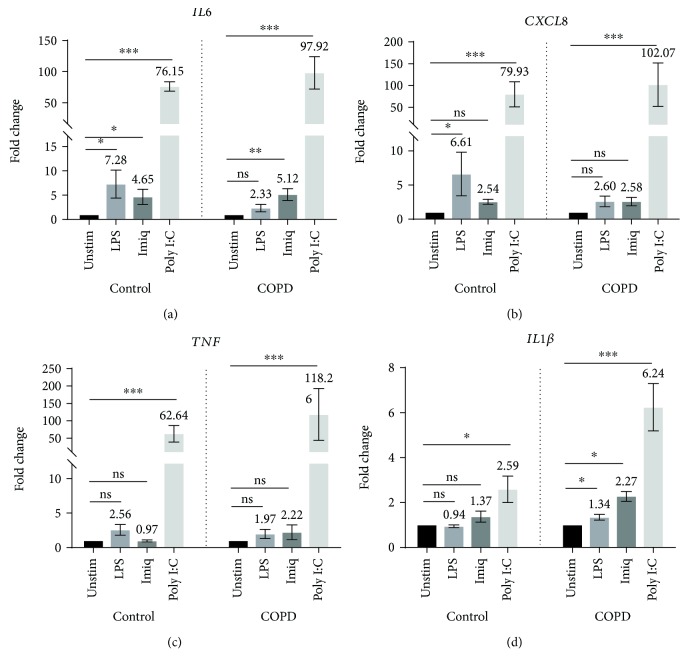
Expression of mRNA for *IL6*, *CXCL8*, *TNF*, and *IL1β* by primary AECs in response to virus-like stimuli (imiquimod or poly I:C) and bacterial LPS. AECs from control subjects (*n* = 7) or COPD patients (*n* = 7) were stimulated with LPS, imiquimod, or poly I:C. Gene expression data (relative to HPRT) were transformed and analyzed by repeated measures one-way ANOVA. For clarity, graphs show fold change relative to unstimulated cells in the corresponding group (control or COPD). Significant differences relative to unstimulated cells are shown as ^∗^*p* < 0.05 and ^∗∗∗^*p* < 0.001. Unstim: unstimulated; Imiq: imiquimod.

As shown in Figure 1, the expression of mRNA for *IL6* was significantly increased by all three stimuli in NAECs and by both virus-like stimuli in CAECs*. CXCL8* and *TNF* were significantly increased by poly I:C, in both populations of cells. Importantly, however, while mRNA expression of *IL1β* was increased in control cells only following stimulation with poly I:C, it was significantly increased by all three stimuli in cells from patients with COPD (*p* < 0.05 for imiquimod and LPS, *p* < 0.01 for poly I:C).

Furthermore, analyzing the fold change from baseline and comparing NAEC with CAEC, *IL1β* stimulation showed a greater response in CAEC (poly I:C *p* = 0.0093; imiquimod *p* = 0.0189; and LPS *p* = 0.0083). For other cytokines, the level of expression in response to LPS, imiquimod, and poly I:C was not significantly greater in COPD cells relative to control cells.

**Figure 2:**
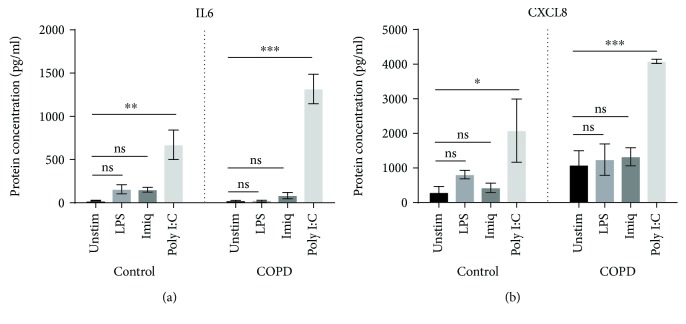
Concentrations of IL6 and CXCL8 protein in culture supernatants of primary AECs in response to virus-like/bacterial stimuli. IL6 and CXCL8 protein were measured using ELISA (*n* = 5). Significant differences are shown as ^∗^*p* < 0.05, ^∗∗^*p* < 0.01, and ^∗∗∗^*p* < 0.001. Unstim: unstimulated; Imiq: imiquimod.

Poly I:C increased concentrations of IL6 and CXCL8, compared to unstimulated cells, in cell culture supernatants of both NAEC and CAEC (Figure 2). Levels of both cytokines were higher in supernatants from CAEC, but while the increased production of IL6 was significantly greater when compared to NAEC (*p* = 0.039), the increase in levels of CXCL8 was not statistically significant (*p* = 0.244). Cell culture supernatants did not contain detectable levels of TNF or IL1*β* protein.

**Figure 3:**
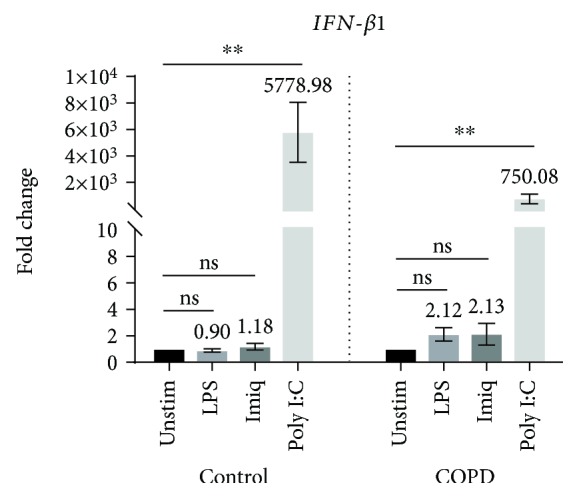
Expression of mRNA for IFNB1 by primary AECs in response to virus-like stimuli and bacterial LPS. Gene expression data (relative to HPRT) were transformed and analyzed by repeated measures one-way ANOVA. Graphs show fold change relative to unstimulated cells in the corresponding group (*n* = 7). Significant differences relative to unstimulated cells are shown as ^∗∗^*p* < 0.01. Unstim: unstimulated; Imiq: imiquimod.

The expression of mRNA for *IFNB1* by NAEC and CAEC was markedly stimulated in response to poly I:C (>1000-fold increase, *p* < 0.001 for both, relative to the corresponding unstimulated cells) (Figure 3). However, there was no significant difference in the levels of expression between the two groups.

### 3.3. Epithelial Responses to Virus-Like/Bacterial Stimuli in a Th1/17-Biased Environment

To investigate the effects of Th1/17 cytokines on these responses, the primary AECs from control subjects (NAECs) were cultured in the presence of the major Th1 cytokine, IFN-*γ*, and the principal Th17 cytokine IL17A. The responses of cells to stimulation with poly I:C, imiquimod, and bacterial LPS were assessed as above.

**Figure 4:**
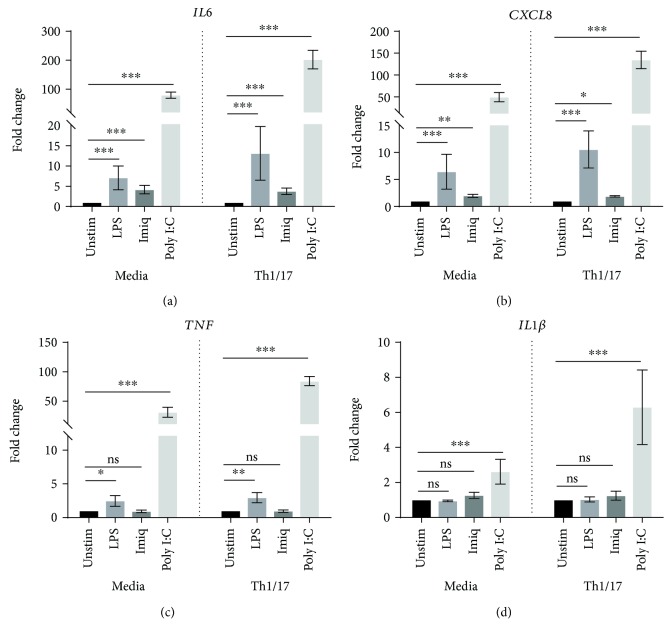
Effect of a Th1/17 environment on the proinflammatory response of primary AECs to virus-like/bacterial stimuli. AECs from control subjects (*n* = 7) were cultured in the absence or presence of Th1/17 cytokines, followed by the stimulation with LPS, imiquimod, or poly I:C. Gene expression data (relative to HPRT) were transformed and analyzed by 2-way ANOVA. Graphs show fold change relative to unstimulated cells in corresponding group (media or Th1/17 environment). The comparison of fold change in cytokine response between the baseline and the Th1/17-biased environment is summarized in Table 4. Significant differences relative to unstimulated cells are shown as ^∗^*p* < 0.05, ^∗∗^*p* < 0.01, and ^∗∗∗^*p* < 0.001. Unstim: unstimulated; Imiq: imiquimod.

Again, poly I:C was the most potent stimulus for increasing the expression of proinflammatory cytokines/chemokines (Figure 4) and interferon-*β*1. Importantly, airway epithelial expression of mRNA for *IL6*, *CXCL8*, *TNF*, and *IL1β* in response to poly I:C was significantly upregulated in a Th1/17 cytokine milieu when compared to the baseline environment (media alone), as summarized in Table 4. *IL6* and *CXCL8* responses to LPS were also significantly enhanced in a Th1/17 environment, relative to media alone. No such effect was observed for imiquimod.

In the Th1/17 environment, significantly upregulated secretion of IL6 in response to poly I:C was demonstrated by assays for protein in culture supernatants (*p* < 0.01) (not shown). CXCL8 secretion was also elevated, but this increase was not statistically significant (not shown).

### 3.4. Modelling the AEC Response in the Inflammatory Cytokine Environment of COPD

The well-differentiated airway epithelial cell line, Calu-3, was used to establish an *in vitro* model of the responses of AECs to virus-like/bacterial stimuli in the Th1/17-biased inflammatory environment of COPD.

**Figure 5:**
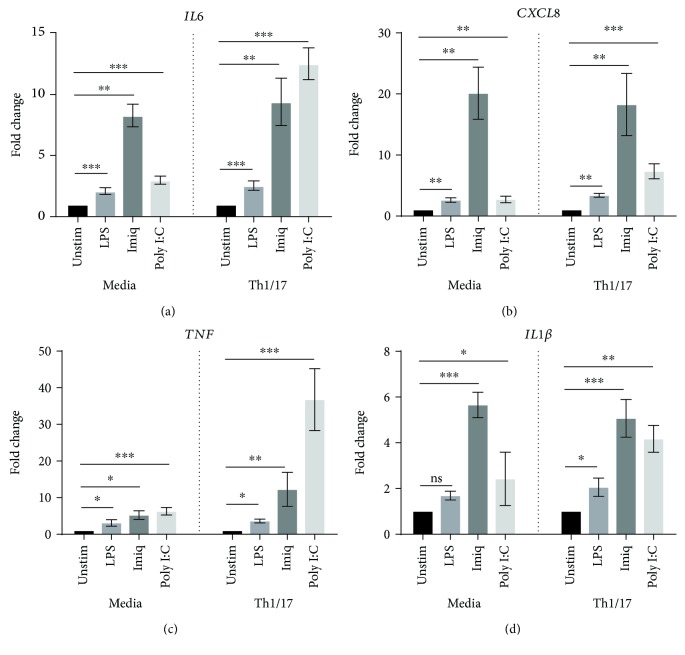
Effect of a Th1/17 environment on expression of mRNA for *IL6*, *CXCL8*, *TNF*, and *IL1β* by Calu-3 cells in response to LPS, imiquimod, or poly I:C. Gene expression data (relative to HPRT) were transformed and analyzed by 2-way ANOVA. Graphs show fold change relative to unstimulated cells in the corresponding group (media or Th1/17 environment) (LPS, imiquimod: *n* = 5; poly I:C: *n* = 6). Significant differences are shown as ^∗^*p* < 0.05, ^∗∗^*p* < 0.01, and ^∗∗∗^*p* < 0.001. Unstim: unstimulated; Imiq: imiquimod.

**Figure 6:**
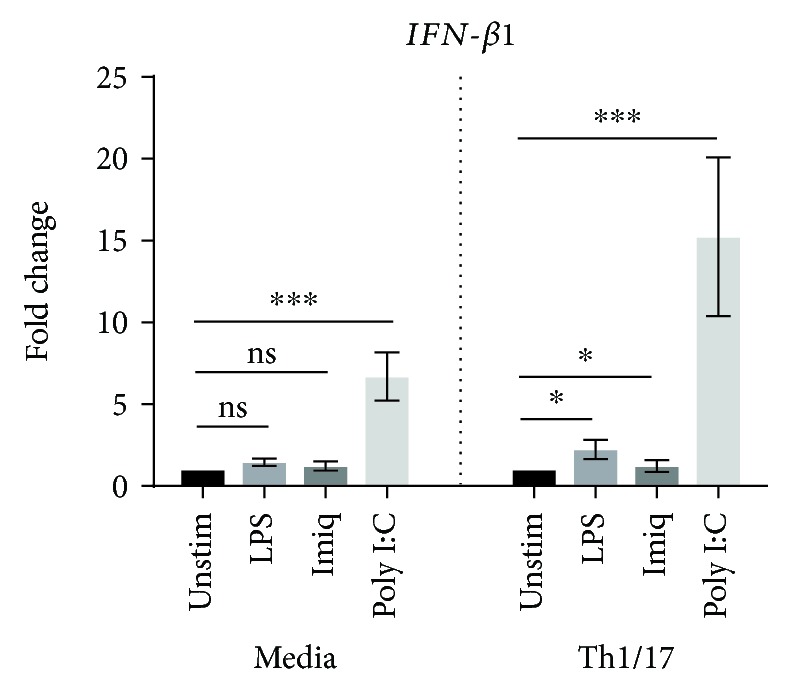
Expression of mRNA for *IFN-β1* by Calu-3 cells pretreated with Th1/17 cytokines, following stimulation with LPS, imiquimod, or poly I:C. *IFN-β1* expression data (relative to HPRT) were transformed and analyzed by 2-way ANOVA (LPS, imiquimod: *n* = 5; poly I:C: *n* = 6). Graphs show fold change relative to unstimulated cells in the corresponding group (media or Th1/17 environment). Significant differences are shown as ^∗^*p* < 0.05, ^∗∗^*p* < 0.01, and ^∗∗∗^*p* < 0.001. Unstim: unstimulated; Imiq: imiquimod.

As shown in Figure 5, all three stimuli were able to significantly increase expression by Calu-3 cells of mRNA for all of the proinflammatory cytokines/chemokines assessed in control media and/or in the Th1/17-biased environment. Imiquimod appeared to be a more effective stimulus for these cells than for primary AECs. Importantly, in the Th1/17 environment, there was significant upregulation of the response to stimulation by poly I:C for *IL6* (*p* < 0.001), *CXCL8* (*p* < 0.01), *TNF* (*p* < 0.001), and *IL1β* (*p* < 0.05). No significant upregulation of mRNA expression was observed in response to imiquimod or LPS. Poly I:C also increased mRNA expression of *IFNB1* by Calu-3 cells, but the magnitude of this response did not change in the Th1/17 environment (Figure 6).

**Figure 7:**
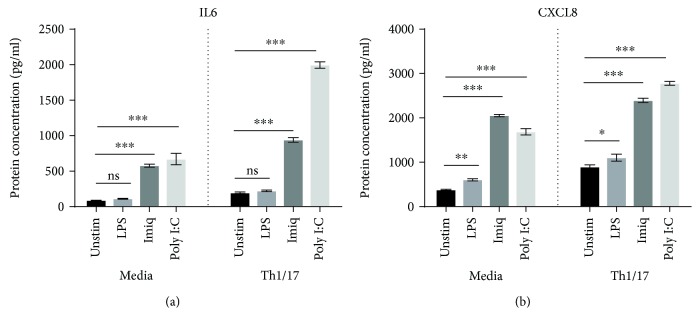
Effect of a Th1/17 environment on IL6 and CXCL8 protein in culture supernatants of Calu-3 cells in response to virus-like/bacterial stimuli. IL6 and CXCL8 protein were measured using ELISA (*n* = 3). Significant differences are shown as ^∗^*p* < 0.05, ^∗∗^*p* < 0.01, and ^∗∗∗^*p* < 0.001. Unstim: unstimulated; Imiq: imiquimod.

Assessment of protein levels in culture supernatants of Calu-3 cells revealed significantly increased concentrations of IL6 and CXCL8 in response to both poly I:C and imiquimod. CXCL8 was also increased in response to LPS, although this stimulus had no significant effect on the secretion of IL6 (Figure 7). Consistent with the mRNA results, in the Th1/17 environment, the levels of secretion of both IL6 and CXCL8 were significantly upregulated, at baseline as well as in response to both poly I:C and imiquimod (*p* < 0.01 for all comparisons). TNF and IL1*β* protein were not detected in Calu-3 cell culture supernatants.

### 3.5. Effect of Th1/17 Cytokine Environment on Expression of Pattern Recognition Receptors

**Figure 8:**
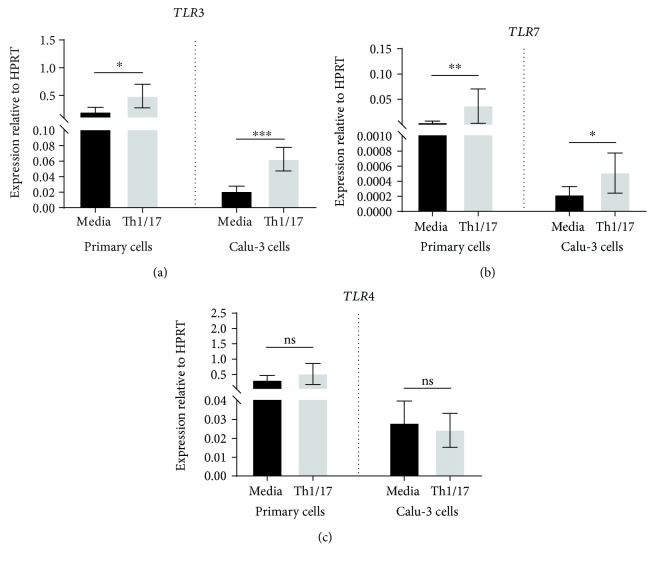
Effect of a Th1/Th17 environment on expression of mRNA for TLRs by primary and Calu-3 airway epithelial cells. Data are shown as expression relative to the control gene HPRT (*n* = 7 for primary AECs; *n* = 13 for Calu-3 cells). Significant differences are shown as ^∗^*p* < 0.05, ^∗∗^*p* < 0.01, and ^∗∗∗^*p* < 0.001.

Expression of mRNA for *TLR3* and *TLR7*, which are toll-like receptors for viruses, was significantly upregulated in NAECs grown in the Th1/17-biased environment (Figure 8). Comparable enhancement of expression was also observed in Calu-3 cells treated with Th1/17 cytokines. In Calu-3 cells, the increase in TLR7 protein was confirmed by western blotting (Supplementary Material file; [Supplementary-material supplementary-material-1]). In contrast, expression of mRNA for *TLR4*, the receptor for bacterial LPS, was not induced by Th1/17 cytokines in either NAECs or Calu-3 cells.

**Figure 9:**
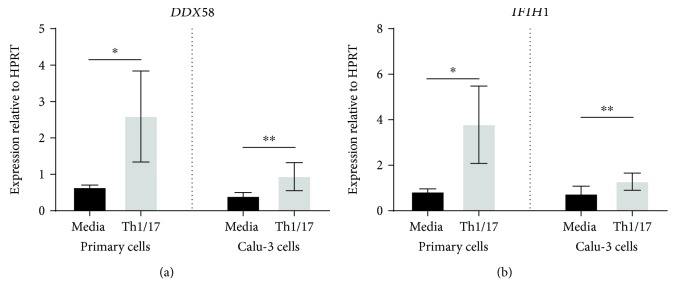
Effect of a Th1/Th17 cytokine environment on expression of mRNA for RLRs by primary and Calu-3 airway epithelial cells. Data are shown as expression relative to the control gene HPRT (*n* = 7 for primary AECs; *n* = 13 for Calu-3 cells). Significant differences are shown as ^∗^*p* < 0.05 and ^∗∗^*p* < 0.01.

Messenger RNA for the RLRs for viruses, *DDX58* and *IFIH1*, was also upregulated by Th1/17 cytokines in both NAECs and Calu-3 AECs (Figure 9).

## 4. Discussion

In this study, we characterized the responses of AEC from patients with cigarette smoke-induced COPD and healthy control subjects to virus-like and bacterial stimuli. The specific contribution of the Th1/17 cytokine environment to changes in these responses was assessed. Furthermore, an *in vitro* model that simulated primary AECs in the Th1/17-biased environment of COPD was established using Calu-3 cells.

The TLR3 ligand poly I:C, a surrogate for viral dsRNA, strongly induced expression of mRNA for a variety of proinflammatory cytokines by AECs from both control subjects and COPD patients. Expression of mRNA for *IFN-β1*, which plays a key role in antiviral responses, was also strongly increased by stimulation with poly I:C. In contrast, the TLR7 agonist imiquimod, another virus-like stimulus, elicited increased expression of mRNA only for *IL6* (in both control and COPD AECs) and *IL1β* (in AECs from COPD patients). Moreover, the magnitude of responses to imiquimod was relatively modest, possibly suggesting a lesser role for the TLR7 pathway in virally induced inflammation in COPD. The bacterial stimulus LPS increased expression of mRNA for *IL6* and *CXCL8* in AECs from control subjects and elevated mRNA level of *IL1β* in cells from COPD patients. Production of IL6 protein by CAEC was also significantly higher than by NAEC, with a borderline increase in levels of CXCL8 protein.

Of note was that in COPD patients, mRNA for *IL1β* was induced by poly I:C, imiquimod, and LPS, with significantly higher levels of expression for all three stimuli, relative to AECs from control subjects. These findings suggest that *IL1B* may play an important role in regulating the response of AECs to airway infections in COPD [10]. However, we were unable to detect similar changes in IL1B protein production. It is possible that IL1B protein is not released into culture supernatant or that levels were below the limit of detection. Further studies using alternate approaches, for example, western blotting of cell lysates, are required to confirm whether production of IL1B in response to these stimuli is enhanced in AEC from patients with COPD.

The Th1/17-biased inflammatory milieu is an important characteristic of COPD, which may alter the airway epithelial cell responses to environmental stimuli, including viruses and bacteria [6, 7]. However, the effect of this biased cytokine environment on epithelial responses is still poorly defined. Our study demonstrated that in a Th1/17 cytokine environment, there was marked enhancement of proinflammatory responses to the viral dsRNA surrogate poly I:C for all cytokines tested, as well as significant enhancement of cytokine responses to bacterial LPS. These findings provide a plausible explanation for the augmented airway epithelial responses to infective stimuli in COPD.

Pattern recognition receptors, located on the AEC plasma membrane and membranes of intracellular vesicles, can detect pathogens by recognizing their conserved molecular structures, which in turn regulate the downstream antiviral and proinflammatory responses. Evidence is emerging to support the involvement of these innate immunity receptors in the pathogenesis of chronic disease of the airways, such as COPD [11] and asthma [12]. This study found that the expression of mRNA for several viral receptors (including *TLR3*, *TLR7*, *DDX58*, and *IFIH1*) was induced in a Th1/17-biased environment, while the bacterial receptor *TLR4* remained unchanged. Enhanced signaling by pattern recognition receptors in the cytokine environment of COPD may contribute to exaggerated inflammatory responses and worsening symptoms in virus-induced exacerbations of disease in these patients. In this context, it is noteworthy that RLRs (e.g., *DDX58* and *IFIH1*) have been shown to be negatively correlated with the FEV_1_ (FEV_1_ % predicted) [13].

Importantly, there were no significant differences in poly I:C-induced increases in expression of mRNA for *IFN-β1* between cells cultured in the absence or presence of Th1/17 cytokines. This suggests that the Th1/17-biased cytokine environment does not itself lead to the impaired antiviral responses that have been associated with COPD [14]. We previously reported analogous findings with respect to the Th2 cytokine environment in asthmatics [9], who also exhibit impairment of antiviral responses. In patients with COPD or asthma, the impaired interferon production might instead be related to factors such as exposure to cigarette smoke [14] or the use of glucocorticoids and bronchodilators, which have been reported to reduce innate antiviral responses [15].

It is possible that results from experiments involving primary AEC may have been affected by hydrocortisone which is included in the standard bronchial epithelial cell growth medium (BEGM). Although this supplement has anti-inflammatory effects, it is commonly used to support growth of AEC and all experimental groups would have been equally affected. However, it is unclear if any of the COPD patients had steroid-insensitive disease.

Further studies of the cellular mechanisms and signaling pathways underlying the effects of the Th1/17 cytokine environment are limited by the availability of primary AECs. Therefore, we sought to establish a cell line model for future studies, because this would be more cost-effective, and would provide a homogeneous population of cells with improved reproducibility of experimental results [16, 17].

Widely available airway epithelial cell lines include Calu-3, A549, BEAS-2B, and 16HBE14o-. Among them, Calu-3 is of particular relevance for studies of pulmonary diseases such as COPD. This well-differentiated cell line is derived from a human lung adenocarcinoma and has been widely used in studies of drug delivery [18, 19], pulmonary drug disposition [20], and pulmonary inflammatory responses [21]. Stewart et al. demonstrated that Calu-3 cells express epithelial markers similar to primary AECs, as well as comparable levels of transepithelial electrical resistance, which reflects the ability of the cells to form tight junction [22]. In addition, Calu-3 cells exhibit secretory activity, reflecting their origin from bronchial submucosal gland cells [23, 24].

In contrast, A549 cells, also derived from a pulmonary adenocarcinoma, typically exhibit the features of type II alveolar cells and are functionally deficient in tight junctions [25]. The bronchial epithelial cell line BEAS-2B was immortalized by infection with hybrid adenovirus 12-simian virus 40, potentially causing abnormal responses to viral and virus-like stimuli [22]. The 16HBE14o- cell line was similarly immortalized with an SV40 plasmid, and although highly differentiated in many respects, it has much lower production of IgA secretory component [26].

We therefore selected Calu-3 cells to model the responses of primary AECs in a Th1/17-biased inflammatory environment. In our experiments, the behavior of these cells was largely similar to that of primary AECs, with increased expression of proinflammatory cytokines in response to all three stimuli and upregulation of expression in the presence of Th1/17 cytokines.

Calu-3 cells also exhibited upregulation of expression of pattern recognition receptors in a Th1/17 cytokine environment, comparable to that of primary AECs. These results suggest that a model based on Calu-3 cells may be useful for further experimental studies of the contribution of AECs, in the Th1/17-biased inflammatory environment of COPD, to the exaggerated inflammatory response in infective exacerbations. Such further work might include identification and targeting of regulatory mechanisms, for example, noncoding RNAs, and the identification of potentially novel therapies controlling the Th1/17-mediated inflammation in COPD [27, 28].

TLR agonists were used to simulate infection with viruses and bacteria in this study. Although the use of these surrogates is widely accepted, the use of live or inactivated pathogens is likely to induce responses of AECs that more closely resemble the *in vivo* situation. Nonetheless, well-characterized surrogates are also useful for studying more specific pathways or biological processes. For example, the poly I:C-stimulated cell model can be used to study the responses through viral dsRNA-activated TLR3 signaling. There are shortcomings in projecting these results to human disease. Only a small number of COPD patients and control subjects can be studied given the complexity of the experiments, there will be genotypic, phenotypic, and environmental differences that cannot be assessed, and a control group with AEC from smokers who do not have COPD would be appropriate, but these are difficult to obtain.

## 5. Conclusion

The results of this study, using primary airway epithelial cells, demonstrate an enhanced response of AECs to viral and bacterial stimuli in cigarette smoke-related COPD. In addition, we developed a novel *in vitro* system using primary AECs from healthy controls and a cell line to investigate the potential contribution of the Th1/17-biased inflammatory environment to these altered responses. The mechanisms by which the Th1/17-biased inflammatory environment promotes proinflammatory responses to infective stimuli appear to include enhanced signaling via pattern recognition receptors. Collectively, the altered expression of cytokines and receptors by airway epithelial cells may help to drive the exaggerated inflammation in acute exacerbations of COPD triggered by viral or bacterial infections.

## Figures and Tables

**Table 1:** Sequence of primers used for qRT-PCR.

Genes	Sequence ID	Forward primers (5′-3′)	Reverse primers (5′-3′)	Product size (bp)
*HPRT1*	NM_000194.3	CAAAGATGGTCAAGGTCGCA	TCAAATCCAACAAAGTCTGGCT	82
*CXCL8*	NM_000584.4	TCAGAGACAGCAGAGCACAC	ACACAGTGAGATGGTTCCTTCC	76
*IL6*	NM_000600.5	AACCTGAACCTTCCAAAGATGG	TCTGGCTTGTTCCTCACTACT	159
*IL1B*	XM_017003988.2	GGACAGGATATGGAGCAACAAGTGG	TTCAACACGCAGGACAGGTACAGAT	125
*IFNB1*	NM_002176.4	GTCACTGTGCCTGGACCATA	AATTGTCCAGTCCCAGAGGC	122
*TNF*	NM_000594.4	TCAGCAAGGACAGCAGAGGA	GTCAGTATGTGAGAGGAAGAGAACC	128
*TLR3*	NM_003265.2	GCTCTGGAAACACGCAAACC	CTCGTCAAAGCCGTTGGACT	97
*TLR4*	NM_138554.5	TGGTGTGTCGGTCCTCAGTGT	GCAGCCAGCAAGAAGCATCAG	91
*TLR7*	NM_016562.4	TCTTGCCTTCTGGAGTTTTTG	CAGTGGTCAGTTGGTTGTGG	150
*IFIH1*	NM_022168.4	GGAGTCAAAGCCCACCATCT	AGACCTTCTTCTGCCACTGTG	146
*DDX58*	NM_014314.4	GCCTTGGCATGTTACACAGC	TTTGGCTTGGGATGTGGTCT	110

TLR: toll-like receptor; IFIH1: interferon-induced helicase C domain-containing protein 1, known as MDA5; DDX58: DExD/H-box helicase 58 (known as RIG-I).

**Table 2:** Performance characteristics of ELISA kits.

Cytokine	Limit of detection	Assay range	Detection method
IL1*β*	3.9 pg/ml	3.9-250 pg/ml	HRP/TMB
IL6	9.4 pg/ml	9.4-600 pg/ml	HRP/TMB
CXCL8	31.2 pg/ml	31.2-2000 pg/ml	HRP/TMB
TNF	15.6 pg/ml	15.6-1000 pg/ml	HRP/TMB

**Table 3:** Subject characteristics.

	Age	Gender	Alcohol	Smoking	Respiratory disease	Medications (related to airway diseases)
Subject 1	60	Female	Yes	Never smoker	N/A	No
Subject 2	48	Male	Yes	Never smoker	N/A	No
Subject 3	59	Female	Yes	Never smoker	N/A	No
Subject 4	57	Male	No	Never smoker	N/A	No
Subject 5	53	Male	Yes	Never smoker	N/A	No
Subject 6	54	Female	Yes	Never smoker	N/A	No
Subject 7	57	Female	Yes	Never smoker	N/A	No
Subject 8	50	Female	Yes	30 years, 1 pack/day	COPD	Albuterol
Subject 9	76	Female	No	30 years, 1 pack/day	COPD	Unknown
Subject 10	53	Male	Yes	27 years, 2 packs/day	COPD	Unknown
Subject 11	66	Male	Yes	48 years, 1 pack/day	COPD	Unknown
Subject 12	53	Male	Yes	20 years, 1 pack/day	COPD	Unknown
Subject 13	62	Female	No	45 years, 1 pack/day	COPD	Albuterol
Subject 14	51	Female	Yes	40 years, 1 pack/day	COPD	Albuterol; ipratropium

**Table 4:** Effects of a Th1/17-biased environment on mRNA responses of primary AECs to virus-like/bacterial stimuli.

Cytokines	LPS	Imiquimod	Poly I:C
*IL6*	↑^∗^	ns	↑^∗^
*CXCL8*	↑^∗^	ns	↑^∗^
*TNF*	ns	ns	↑^∗^
*IL1β*	ns	ns	↑^∗∗∗^
*IFN-β1*	ns	ns	ns

Table 4 summarizes the change in gene expression in AEC cultured in the presence of Th1/17 cytokines, relative to cells grown in media alone, following stimulation with LPS, imiquimod, and poly I:C, respectively (*n* = 7 for each group). The significant differences compared to cells cultured in media alone are shown as ^∗^*p* < 0.05 and ^∗∗∗^*p* < 0.001. For example, first “↑^∗^” in the table represents that the increase in the expression of mRNA for IL6 in response to LPS was significantly greater in AEC cultured in a Th1/17-biased environment than the increase in the expression of mRNA for IL6 in response to LPS in AECs grown in the absence of Th1/17 cytokines.

## Data Availability

The data used to support the findings of this study are available from the corresponding author upon request.

## Supplementary Materials

Supplementary Materials Supplementary Figure 1: expression of mRNA for IL6, IL8, and IL1*β* by primary AEC in response to increasing concentrations of IFN-*γ* and IL17A. Supplementary Figure 2: expression of mRNA for IL6, IL8, and TNF-*α* by primary AEC following stimulation with increasing concentrations of poly I:C. Supplementary Figure 3: expression of mRNA for IL6 and IL8 by primary AEC following stimulation with increasing concentrations of imiquimod. Supplementary Figure 4: expression of mRNA for IL6 and IL8 by primary AEC following stimulation with increasing concentrations of LPS. Supplementary Figure 5: expression of mRNA for CXCL10 and IL8 by Calu-3 cells in response to increasing concentrations of IFN-*γ* and IL17A. Supplementary Figure 6: expression of mRNA for IL8 and IL6 by Calu-3 cells in response to increasing concentrations of LPS. Supplementary Table 1: thermocycler conditions. Supplementary Figure 7: TLR7 protein expression was increased in Calu-3 cells grown in a Th1/17-biased environment.
