# Supplementary material for: Th1/17-Biased Inflammatory Environment Associated with COPD Alters the Response of Airway Epithelial Cells to Viral and Bacterial Stimuli
Source: Mediators Inflamm. 2019 Aug 25;2019:7281462. doi: 10.1155/2019/7281462 (PMC6732592; doi:10.1155/2019/7281462)
Supplement: Supplementary Materials — Supplementary Figure 1: expression of mRNA for IL6, IL8, and IL1β by primary AEC in response to increasing concentrations of IFN-γ and IL17A. Supplementary Figure 2: expression of mRNA for IL6, IL8, and TNF-α by primary AEC following stimulation with increasing concentrations of poly I:C. Supplementary Figure 3: expression of mRNA for IL6 and IL8 by primary AEC following stimulation with increasing concentrations of imiquimod. Supplementary Figure 4: expression of mRNA for IL6 and IL8 by primary AEC following stimulation with increasing concentrations of LPS. Supplementary Figure 5: expression of mRNA for CXCL10 and IL8 by Calu-3 cells in response to increasing concentrations of IFN-γ and IL17A. Supplementary Figure 6: expression of mRNA for IL8 and IL6 by Calu-3 cells in response to increasing concentrations of LPS. Supplementary Table 1: thermocycler conditions. Supplementary Figure 7: TLR7 protein expression was increased in Calu-3 cells grown in a Th1/17-biased environment. [file 7281462.f1.pdf]

Supplementary Materials

1 Preliminary titration experiments

For both primary AEC and Calu-3 cells, the concentrations of IFN-γ and IL-17A used for pre-treatment and the concentrations of each stimulus (poly I:C, LPS and imiquimod) were selected based on preliminary titration experiments.

1.1 Determination of the concentration of Th1/17 cytokines for pre-treatment of primary AEC

A concentration of 6 ng/ml for both IFN-γ and IL-17A was selected for pre-treatment of primary AECs. These was the highest concentration that did not result in a significant increase in mRNA for a variety of pro-inflammatory cytokines (IL-6, IL-8 and IL-1β) relative to cells in media alone (Supplementary Figure 1).

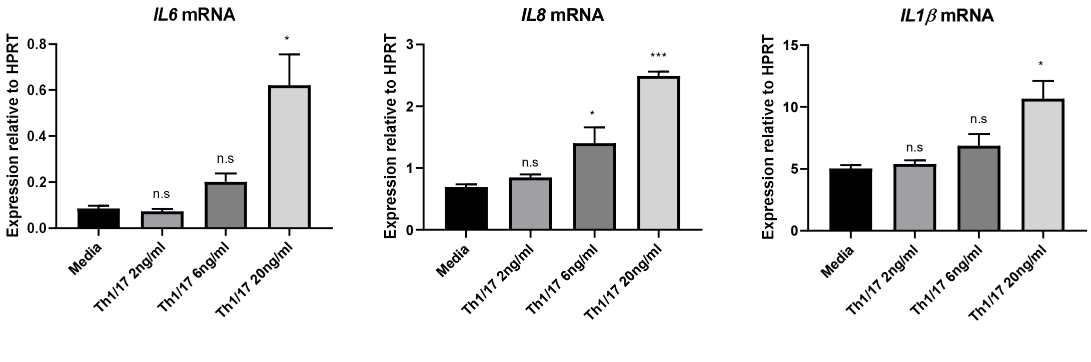

Supplementary Figure 1: Expression of mRNA for IL-6, IL-8 and IL-1β by primary AEC in response to increasing concentrations of IFN-γ and IL-17A. Significant

differences relative to cells grown in media alone are shown as * ($p<0.05$) and *** ($p<0.001$).

1.2 Determination of the concentration of stimuli for experiments with primary AECs

In primary AEC, a concentration of 1 µg/ml of poly I:C was selected for stimulation experiments. This concentration was the lowest concentration tested that consistently resulted in significant increases in mRNA for pro-inflammatory cytokines (IL-6, IL-8 and TNF-α) after stimulation for 4 hours relative to cells in media alone (Supplementary Figure 2).

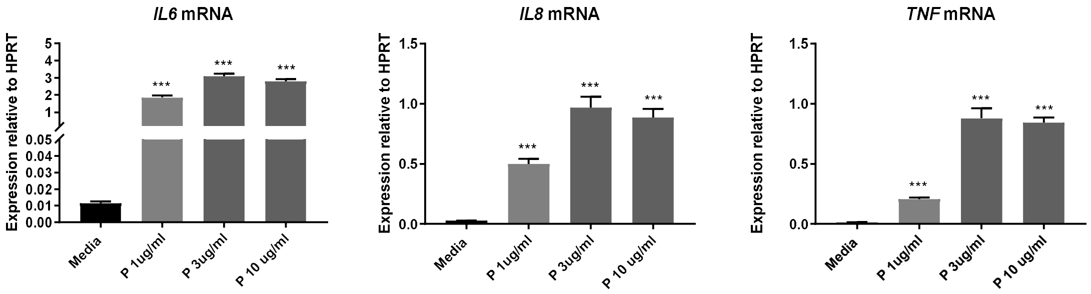

Supplementary Figure 2: Expression of mRNA for IL-6, IL-8 and TNF-α by primary AEC following stimulation with increasing concentrations of poly I:C. Significant differences relative to unstimulated cells (media) are shown as *** ($p<0.001$).

In primary AEC a concentration of 7 µg/ml of imiquimod was selected for stimulation experiments. This concentration was the lowest concentration tested that consistently resulted in significant increases in mRNA for a variety of pro-inflammatory cytokines (IL-6 and IL-8) after stimulation for 4 hours relative to cells in media alone (Supplementary Figure 3).

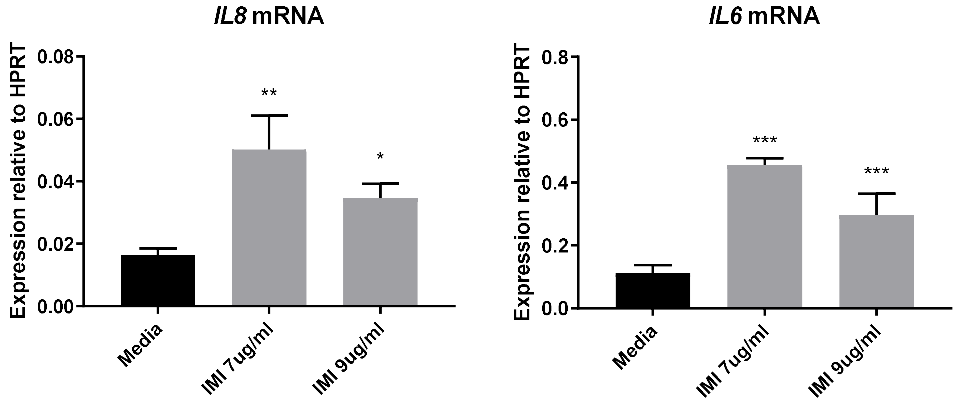

Supplementary Figure 3: Expression of mRNA for IL-6 and IL-8 by primary AEC following stimulation with increasing concentrations of imiquimod. Significant differences relative to unstimulated cells (media) are shown as * (p<0.05), ** (p<0.01) and *** (p<0.001).

In primary AEC a concentration of 1.5 μg/ml of LPS was selected for stimulation experiments. This concentration was the lowest concentration tested that consistently resulted in noticeable increases in mRNA for a variety of pro-inflammatory cytokines (IL-6 and IL-8) after stimulation for 4 hours relative to cells in media alone (Supplementary Figure 4).

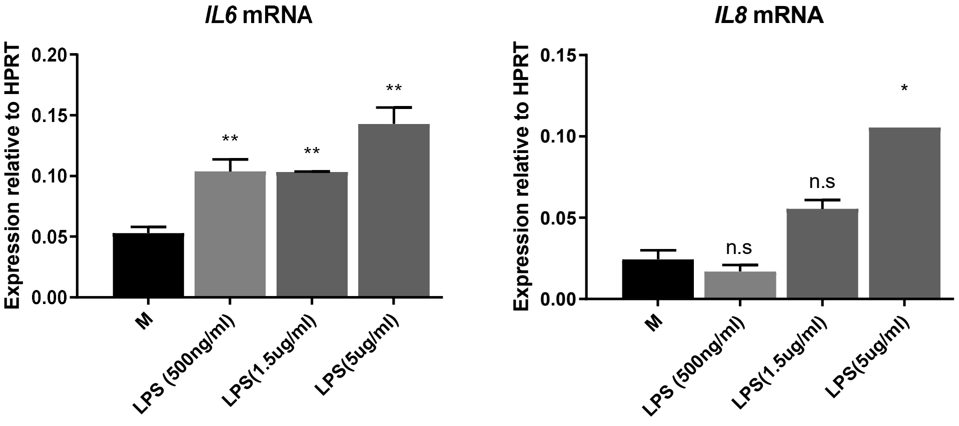

Supplementary Figure 4: Expression of mRNA for IL-6 and IL-8 by primary AEC following stimulation with increasing concentrations of LPS. Significant differences relative to unstimulated cells (media) are shown as * ($p<0.05$) and ** ($p<0.01$).

1.3 Determination of the concentration of Th1/17 cytokines for pre-treatment of Calu-3 cells

A concentration of 2 ng/ml for both IFN-γ and IL-17A LPS was selected for pre-treatment of Calu-3 cells. This was the concentration just below the concentration that resulted in a significant increases in mRNA for a variety of pro-inflammatory cytokines (IL-8 and CXCL10) relative to cells grown in media alone (Supplementary Figure 5).

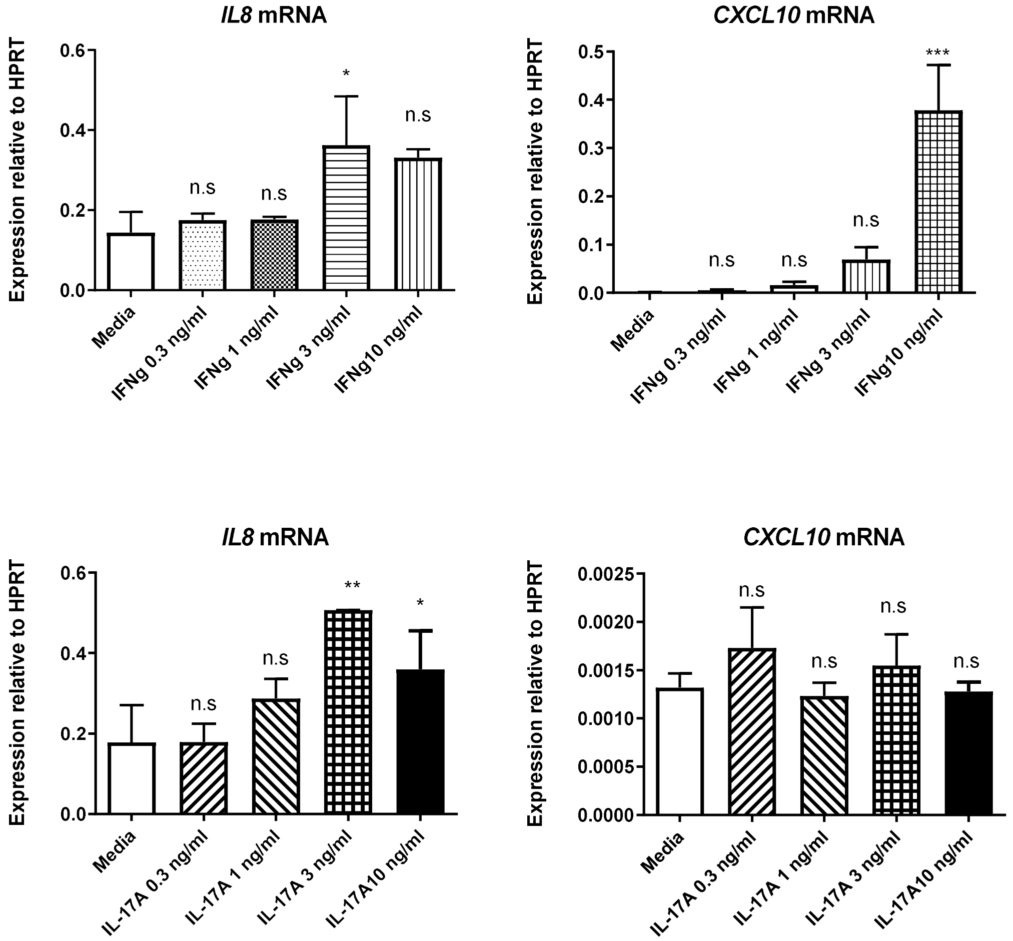

Supplementary Figure 5: Expression of mRNA for CXCL10 and IL8 by Calu-3 cells in response to increasing concentrations of IFN-γ and IL-17A. Significant differences relative to cells grown in media alone are shown as * (p<0.05) ** (p<0.001) and *** (p<0.*01).

1.4 Determination of the concentration of stimuli for experiments with Calu-3 cells

The concentration of poly I:C and imiquimod used to stimulate Calu-3 cells was determined in previously published work from our laboratory [1].

A concentration of 500 ng/ml of LPS was selected to stimulate Calu-3 cells. This concentration was the lowest concentration that elicited a noticeable increase in mRNA for IL-8 and IL-6 after stimulation for 4 hours relative to cells in media alone (Supplementary Figure 6):

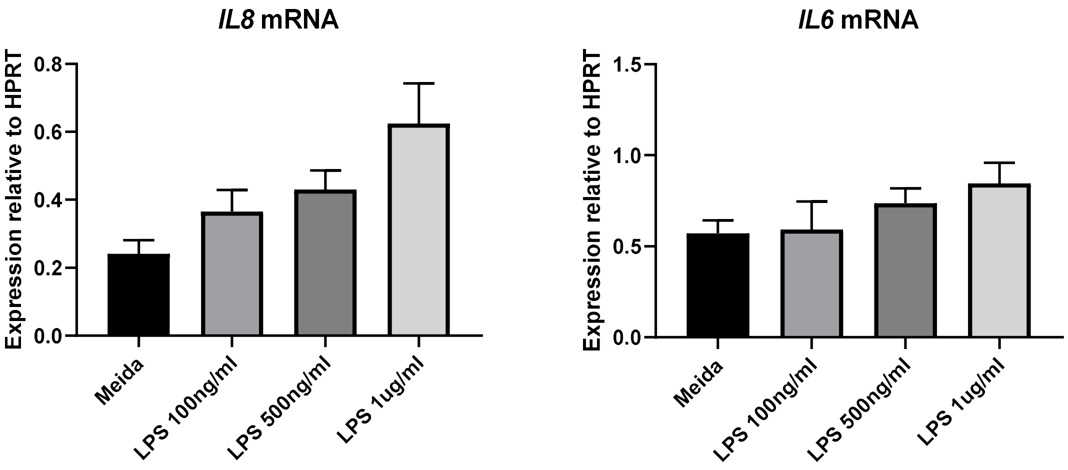

Supplementary Figure 6: Expression of mRNA for IL8 and IL-6 by Calu-3 cells in response to increasing concentrations of LPS.

2 Supplementary materials and methods

These supplementary methods describe the RNA isolation, cDNA synthesis and real-time PCR in greater detail.

2.1 RNA extraction

Cells were scraped from plates using a cell-scraper (Greiner Bio-one, VIC, Australia) and lysed in 1ml TRI reagent (Sigma-Aldrich). Cell samples were then transferred to a 1.5 ml microcentrifuge tube (Life Technologies, VIC, Australia). 200 µL of chloroform (Ajax Finechem

Pty Ltd, NSW, Australia) was added to each tube and vortexed samples for up to 30 seconds. Samples were kept at room temperature for 15 minutes and then centrifuged at 14,000 g for 15 minutes. The upper aqueous layer was carefully removed and transferred this to a new tube. The bottom layer was discarded as chemical waste. 500 µL isopropanol (Sigma-Aldrich) and 8 µL of 5 mg/ml glycogen (Sigma-Aldrich) were added to the same tube, after which the samples were vortexed and left at room temperature for 10 minutes. Samples were then, centrifuged at 14,000 g and 4 °C for 10 minutes.

The supernatant was discarded (taking care not to lose the pellet), and 1 ml of 80% ethanol in DEPC water was added to the pellet and vortexed briefly to wash. Samples were centrifuged at 7500 g for 5 minutes (room temperature), and the supernatant and all residual ethanol was removed and discarded. After drying the pellets briefly using a vacuum pump, the pellets were resuspended in a small volume of DEPC-treated water (20 µL). The concentration and A260/280 ratio of the RNA samples was measured using a Nano-drop spectrophotometer. RNA samples with a purity ratio (A260/A280) of 1.8~2 were considered as acceptable. Lastly, the tubes were labelled and stored them at -20 °C (short-term) or -80 °C (longer-term).

2.2 DNase Treatment and Reverse Transcription

To perform the DNase treatment, 1 µL Turbo DNase (Life Technologies) and 0.5 µL 10X Turbo DNase buffer (Life Technologies) were added to each 200 µL tube (Life Technologies), followed by adding 8.5 µL total RNA sample in the same tube. Tubes were vortexed and briefly spun down. Tubes were then, incubated at 37 °C for 30 minutes. After incubation, 1 µL of 50 mM EDTA was added to each tube. Tubes were vortexed and spun down before being incubated at 75 °C for 10 minutes.

In a new 200 µL tube, 10 µL DNase-treated RNA, 1 µL Oligo dT primer (Rocha, NSW, Australia) and 2 µL Random hexamer primers (Roche) were mixed well and heated to 65 °C for 5 min, then immediately transferred to ice for 5 min.

RT master mix was prepared by mixing 4 µL 5X RT buffer (Roche), 0.5 µL RNase inhibitor (Roche), 2 µL dNTP mix (Roche) and 0.5 µL Transcriptor enzyme (Roche) for each sample. 7 µL of RT master mix was added to each sample tube, mixed well and spun down. Samples were incubated at 25 °C for 10 min, 55 °C for 30 min, then inactivated by heating to 85 °C for 5 min, then placed the samples on ice. RT products were diluted with distilled water and stored at -20 °C until use.

2.3 Quantitative real-time RT- PCR

Expression of mRNA was assessed using qRT-PCR with SensiFast SYBR green (BioLine, Tauton, MA, USA). Primers used for qRT-PCR were designed in-house (Primer Premier 6 software and Primer3) and synthesized by Integrated DNA Technologies (Illinois, USA) (**Table 1**). Specifically, 10µl Sensi-fast, 7 µl RNase/DNase-free water, 0.5 µl forward and reverse PCR primers were mixed into the appropriate wells of a 96 well PCR plate. 2 µl of cDNA was then added to the wells containing the master mix for the gene of interest.

Reactions were performed using a Roche LightCycler 480 system (Roche Diagnostics, Indianapolis, IN, USA), with gene expression normalized to the housekeeping-gene hypoxanthine-guanine phosphoribosyl transferase (HPRT). The thermocycler conditions are outline in **Supplementary Table 1**.

Supplementary Table 1. Thermocycler conditions

**Stage**		**Temp**	**Time**
Pre-incubation	Hold	95°C	2 min
Amplification	Cycle (45 Cycles)	95°C	5 sec
		60°C	15 sec
Melting curve	Hold	95°C	5 sec
	Hold	65°C	1 min
	Continuing	97°C	0.11 °C/sec
Cooling	Hold	40°C	30s

Data were expressed relative to *HPRT* using the ΔCt method. The following equation, which is recommended by Applied Biosystems, was used to calculate relative gene expression:

Target/HPRT= $2^{-\Delta ct}$; where ΔCt = Ct (Target) – Ct (HPRT)

3 Western blot for TLR7

Western blotting confirmed there was a significant increase in TLR7 protein when Calu-3 cells were grown in the presence of Th1/Th17 cytokines (Supplementary Figure 7).

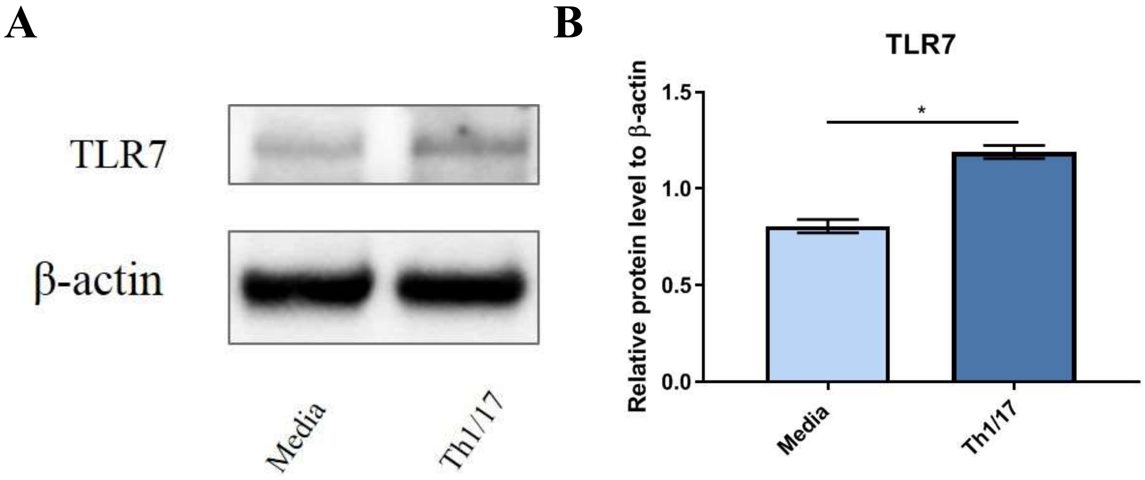

Supplementary Figure 7: TLR7 protein expression was increased in Calu-3 cells grown in a Th1/17-biased environment. Protein production was assessed using Western blot (3 independent experiments). Relative levels of TLR7 protein are expressed as mean ± S.E.M. Significant differences are shown as * ($p<0.05$).

4 Supplementary References

1. Herbert, C., et al., *Allergic environment enhances airway epithelial pro-inflammatory responses to rhinovirus infection.* Clin Sci (Lond), 2017. **131**(6): p. 499-509.
